# Lower Arm Muscle Activation during Indirect-Localized Vibration: The Influence of Skill Levels When Applying Different Acceleration Loads

**DOI:** 10.3389/fphys.2016.00242

**Published:** 2016-06-16

**Authors:** Johnny Padulo, Riccardo Di Giminiani, Antonio Dello Iacono, Alessandro M. Zagatto, Gian M. Migliaccio, Zoran Grgantov, Luca P. Ardigò

**Affiliations:** ^1^University eCampusNovedrate, Italy; ^2^Faculty of Kinesiology, University of SplitSplit, Croatia; ^3^Department of Biotechnological and Applied Clinical Sciences, University of L'AquilaL'Aquila, Italy; ^4^Zinman College of Physical Education and Sport Sciences, Wingate InstituteNetanya, Israel; ^5^Department of Physical Education, Faculty of Sciences, Univ Estadual Paulista - UNESPBauru, Brazil; ^6^Performance Research, Sport Science LabLondon, UK; ^7^Department of Neurosciences, Biomedicine and Movement Sciences, School of Exercise and Sport Science, University of VeronaVerona, Italy

**Keywords:** muscle contraction, motor behavior, fatigue, vibration, sports

## Abstract

We investigated the electromyographic response to synchronous indirect-localized vibration interventions in international and national table tennis players. Twenty-six male table tennis players, in a standing position, underwent firstly an upper arms maximal voluntary contraction and thereafter two different 30-s vibration interventions in random order: high acceleration load (peak acceleration = 12.8 g, frequency = 40 Hz; peak-to-peak displacement = 4.0 mm), and low acceleration load (peak acceleration = 7.2 g, frequency = 30 Hz, peak-to-peak displacement = 4.0 mm). Surface electromyography root mean square from *brachioradialis, extensor digitorum, flexor carpi radialis*, and *flexor digitorum superficialis* recorded during the two vibration interventions was normalized to the maximal voluntary contraction recording. Normalized surface electromyography root mean square was higher in international table tennis players with respect to national ones in all the interactions between muscles and vibration conditions (*P* < 0.05), with the exception of *flexor carpi radialis* (at low acceleration load, *P* > 0.05). The difference in normalized surface electromyography root mean square between international table tennis players and national ones increased in all the muscles with high acceleration load (*P* < 0.05), with the exception of *flexor digitorum superficialis* (*P* > 0.05). The muscle activation during indirect-localized vibration seems to be both skill level and muscle dependent. These results can optimize the training intervention in table tennis players when applying indirect-localized vibration to lower arm muscles. Future investigations should discriminate between middle- and long-term adaptations in response to specific vibration loads.

## Introduction

In table tennis (TT) players, both motor skills and bioenergetics capacities are crucial in discriminating the player's level (Mori et al., 2002; Padulo et al., 2016). Particularly, a high skill level is characterized by fast and precise movements (Yarrow et al., 2009). Yet, high-level players have higher maximal oxygen consumption and/or anaerobic/aerobic performance indicators as well (Zagatto and Gobatto, 2012). Overall, they consequently achieve higher performance in terms of scores. Not surprisingly, the low-level athlete can compete with the high-level one for only a few minutes, because the novice is not able to keep up with the opponent's pace due to his/her poorer timing capability (Ak and Kocak, 2010). In fact, a TT ball travels at ~27 m·s^−1^ during the game and TT players have only ~1 s to prepare for the return after the opponent hits the ball (Ak and Kocak, 2010). Moreover, the overall superiority of the highest-level athletes becomes even more evident under fatigued conditions (Aune et al., 2008), especially in terms of fewer errors made (Padulo et al., 2016).

The ability to be fast and precise (in other words, “less variable”; Aune et al., 2008) during a performance requires superior neuromuscular control of movement, especially in the upper limbs (Bootsma et al., 2010), and also when considering the 100÷200-ms visuomotor delay (Van Soest et al., 1994). Contextually, in these intermittent activities performance may be limited either by potential mechanisms occurring within cortical regions or due to the neuromuscular properties of the peripheral contractile elements (Enoka, 2002; Girard and Millet, 2009).

The discrete movements of TT players across different joints (i.e., hand and finger) are controlled by the primary motor cortex, which is organized into highly interconnected neural assemblies (localized groups of neurons with similar inputs and outputs; Adkins et al., 2006). The coordinated activation of these neural assemblies then encodes complex and multi-joint movements. The new movement sequences during skill learning are acquired and refined with the involvement of changes in the connectivity between these neural structures, i.e., the motor cortex plasticity. The motor cortex plasticity is reflected as an alteration in the cortical synapse number, synaptic strength, and the topography of stimulation-evoked movement representations (Adkins et al., 2006). For example, highly skilled racket players have larger hand motor representation and enhanced motor-evoked potentials (MEP) compared with less proficient players and non-playing controls (Pearce et al., 2000). In synthesis, the neural processes of skill performance that are relevant to a discussion of the differences and similarities in brain activity among elite and expert athletes can be grouped into these four neural process: neural effectiveness, cortical expansion, specialized processing, and internal models (Callan and Naito, 2014).

In this connection, the vibration intervention (vibration applied directly to a muscle) applied during a voluntary contraction can induce prolonged changes in the excitatory/inhibitory state of the primary motor cortex, mediated by Ia fibers afferent input (Rollnik et al., 2001; Marconi et al., 2008). In fact, muscle vibration is a strong proprioceptive *stimulus* that reaches both the primary somatosensory and the motor cortices directly (Rollnik et al., 2001; Marconi et al., 2008). In addition, other experiments carried out on the muscle-tendon unit indicated that vibration exerts a frequency-dependent effect on the Ia afferent firing rate, which is reflected in differential frequency-dependent effects on corticospinal motor neuron excitability (Steyvers et al., 2003). Overall, these results demonstrate that the modulation in corticospinal excitability is depending on the magnitude of the vibration stimulus (i.e., the acceleration) when it is applied directly on the muscle-tendon complex. Additionally, considering that the skill level of athletes is characterized by specific neural processes (Callan and Naito, 2014), the presence of a skill-dependent activation strategy (Basmajian, 1977), mediated by this proprioceptive stimulus (vibration), could be expected among athletes of different skill level. In other words, the vibratory effect (in terms of muscle response) could be dependent on a selective modulation of proprioceptive integration in the motor cortex, that in its turn is related to the skill level (Rosenkranz and Rothwell, 2012). Additionally, the selective recruitment of motor units and synchronization during vibration (Pollock et al., 2012) can occur among athletes of different skill level (Enoka, 1997).

Therefore, when indirect vibration (i.e., whole-body, cable and dumbbell) is applied, we hypothesize that it can influence positively (i.e., increase) the amplitude of the electromyographic activity in the lower arm muscles (Cardinale and Bosco, 2003; Felici, 2006; Rittweger, 2010). This vibration effect would be related to the interaction between the skill level and the magnitude of vibration.

The aim of the present study was to investigate the surface electromyographic response to synchronous localized-indirect vibration (by means of a dumbbell) of different vibration loads and in relation to the skill level of TT players.

## Materials and methods

### Participants

Twenty-six (three left-handed) male TT players participated in the study. All players were divided into two groups according to their best performance and national/international ranking within the Italian Table Tennis Federation. Fourteen players were assigned to the international level (HL) group and the remaining 12 to the national level (LL) group. HL participants' anthropometric data were: age 23.6 ± 3.2 y; mass 63.1 ± 4.5 kg; height 1.72 ± 0.08 m; body mass index (BMI) 21.36 ± 1.54 kg·m^−2^. LL participants' anthropometric data were: age 30.7 ± 3.7 y; mass 76.7 ± 4.3 kg; height 1.75 ± 0.04 m; BMI 25.07 ± 0.82 kg·m^−2^. The inclusion criteria were: (1) high skill with 15 years of experience in TT training and competitions, and having taken part in international (HL) or national (LL) Championships at the time of the investigation, (2) having regularly competed during the previous competitive season, and (3) possession of a valid medical clearance for competition. The subjects were healthy, without any muscular, neurological, and/or tendinous injury, and were not taking any medications. Each subject was informed of the procedures, methods, benefits, and possible risks of the study. Informed consent for experimentation with human subjects was obtained from all individual participants included in the study. All procedures performed in the study involving human participants were in accordance with the ethical standards of the institutional research committee and with the 1964 Helsinki declaration and its later amendments, or with comparable ethical standards.

Subjects refrained from drinking alcohol or caffeinated beverages for 24 h before testing, and fasted for at least 4 h prior to visiting the laboratory. Each subject completed all trials at the same time on the testing days to eliminate any influence of circadian variation (Atkinson and Reilly, 1996). Both groups were homogeneous with regard to training *status*. None of the subjects underwent any strenuous endurance activity and/or resistance training outside the usual training protocol.

### Protocol

All the participants were in good health and they all carried out the test during the same period of the sport season. The relevant data were collected during a single session (one day), from 3 to 6 p.m. to avoid circadian variations (Ammar et al., 2015), under an average temperature of 23°C (min 22°C, max 24°C). Four participants (1 HL and 3 LL) were identified as left-handed after being asked their preferred hand used for TT. Each subject wore typical TT sportswear. All the experiments were carried out in the “Table Tennis International Centre.”

### Electromyography analysis and maximal contraction

**Figure 1:**
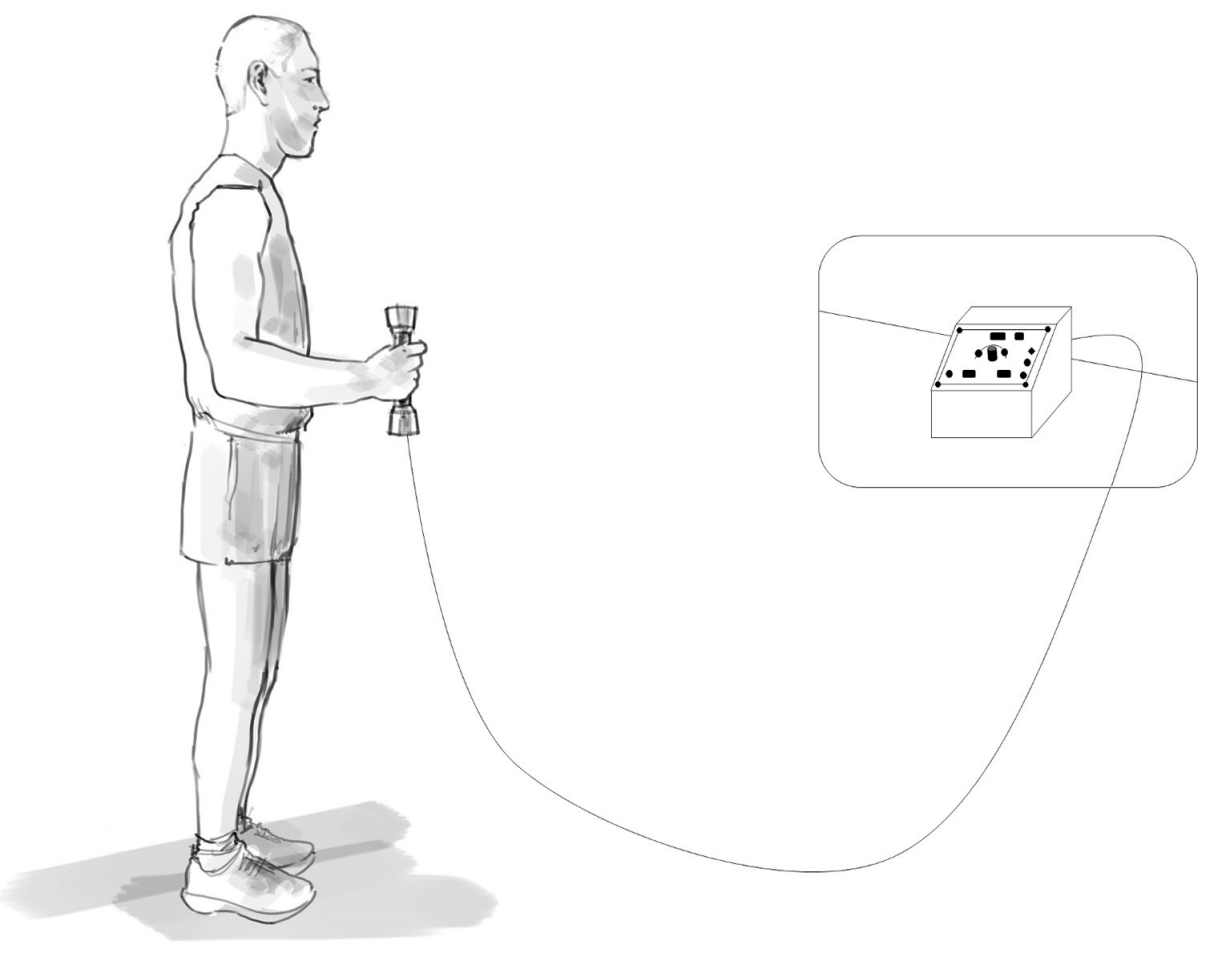
**Schematic sketch of the subject's posture for maximal isometric voluntary contraction and vibration interventions**.

The surface electromyographic responses (sEMG) of dominant arm *brachioradialis* (BR), *extensor digitorum* (ED), *flexor carpi radialis* (FC), and *flexor digitorum superficialis* (FS) were recorded during the two vibration interventions. Subjects were asked to contract their muscles against manual resistance to let the belly of the muscle be palpated by the operator. Bipolar (Ambu Blu Sensor, Ballerup, Denmark) surface electrodes (inter-electrode distance 0.5 cm) were fixed longitudinally over the marked muscle belly and located according to the recommendations of SENIAM (Hermens et al., 2000). Prior to applying the gel-coated electrodes, the skin was shaved and cleansed with alcohol to minimize impedance (<10 kω). To prevent motion artifacts, cables were secured using elastic bands (Vetrap, 3M Italia, Pioltello, Italy; Padulo et al., 2013a). The elbow angle was monitored by using an electrogoniometer connected to the MuscleLab (Ergotest Innovation, Langensund, Norway). Additionally, the Muscle-Lab software converted the amplified sEMG raw signal (an amplifier [gain setting 100 Hz; input impedance 1000 ω; 2 GV common mode rejection rate 100 dB; input noise level within 1 kHz band 3 mVcc], and a Butterworth band-pass filter [3-dB low cut-off frequency 8 Hz; 3-dB high cut-off frequency 1200 Hz]) *via* its hardware in root mean square (sEMG_rms_, mV; Fukuda et al., 2010), with a signal total error ± 0.5%. Then, sEMG_rms_ was expressed as a function of time and averaged for each vibration intervention (30 s) for further analysis. One hour prior to testing, maximal isometric voluntary contractions (MVC) were performed on the vibrating device dumbbell for sEMG normalization (Duc et al., 2008; Padulo et al., 2013b). The vibrating device dumbbell weighs 1480 g. MVC was determined with each subject standing with the arm semi-flexed at 90° (elbow angle) and the hand in a neutral position while gripping the dumbbell (Figure 1). Each subject performed four attempts and the best was selected for analysis (Mischi et al., 2010).

### Lower arm muscles and vibration

The vibrating device used for the study was the Nemes (Bosco System, Langensund, Norway). In the high acceleration load (**HAL**) intervention a_Peak_ was equal to 12.8 g, *f* = 40 Hz, and *D* = 4.0 mm, whereas in the low acceleration load (LAL) the a_Peak_ was equal to 7.2 g, *f* = 30 Hz, and *D* = 4.0 mm. Each of the two interventions lasted 30 s, separated by 5 min of recovery (in randomized order; Bosco et al., 1999). During the vibration interventions the participants assumed the same position adopted for sEMG normalization—in the standing position in front of the device with the dominant arm semi-flexed at 90°, while in the neutral position gripping the dumbbell (Figure 1). About dumbbell grip force, participants were given the following instruction: “just hold it.”

### Statistical analysis

All the data are reported as the mean ± standard deviation (SD). Assumption of normality was verified by using the Shapiro-Wilk test. Maximal isometric voluntary contractions normalized sEMG (sEMG_norm_) values in the BR, ED, FC, and FS were analysed by using a two-way ANOVA with repeated measures and the Bonferroni *post-hoc* test to adjust the *P*-value in relation to the number of contrasts that were performed. The within factor was the vibration intervention (2 levels: HAL and LAL), whereas the between factor was the TT level (2 levels: HL and LL). A *t*-test was used to compare HAL and LAL for each muscle (BR, ED, FC, and FS). To assess the variability of the sEMG_norm_ measures, we calculated their coefficient of variation (CV = SD/mean, %). The effect size (ES) was calculated with eta squared (η^2^) and classified as small (0.01 < η^2^ < 0.06) medium (0.06 < η^2^ < 0.146) and large (η^2^ > 0.14) (Cohen, 1988). Significance level was set at *P* ≤ 0.05 and the statistical analyses were performed by using SPSS (version 15.00).

## Results

**Figure 2:**
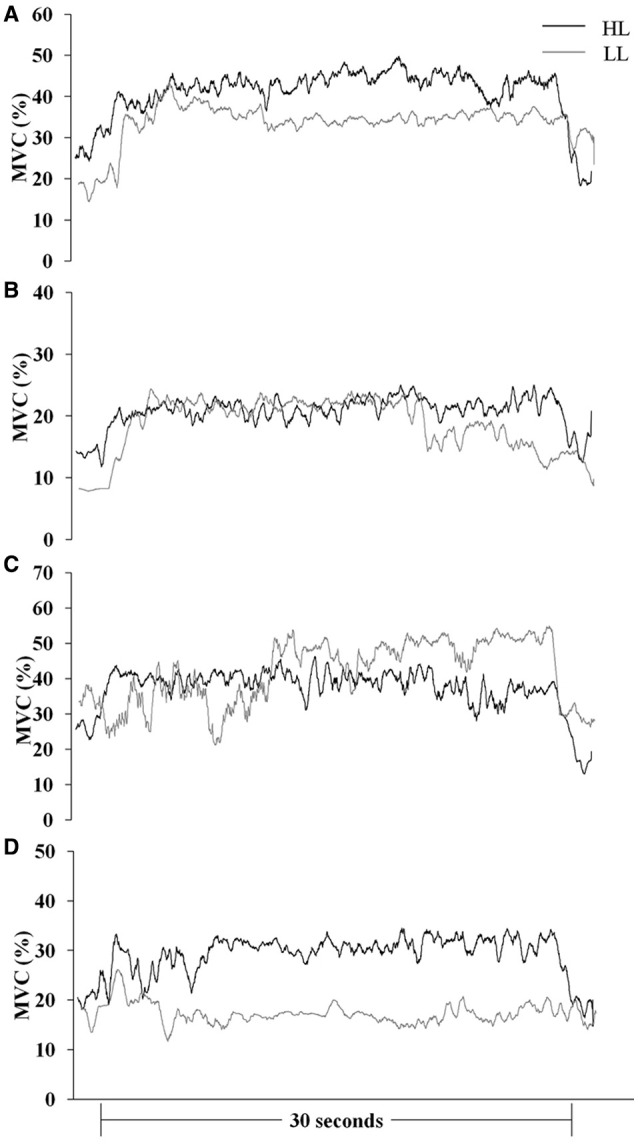
**Mean MVC-normalized surface electromyography root mean square (MVC %) recording over time during the LAL vibration intervention in international (HL)- and national (LL)-level groups. (A)** *brachioradialis*, **(B)** *extensor digitorum*, **(C)** *flexor carpi radialis*, and **(D)** *flexor digitorum superficialis*.

**Figure 3:**
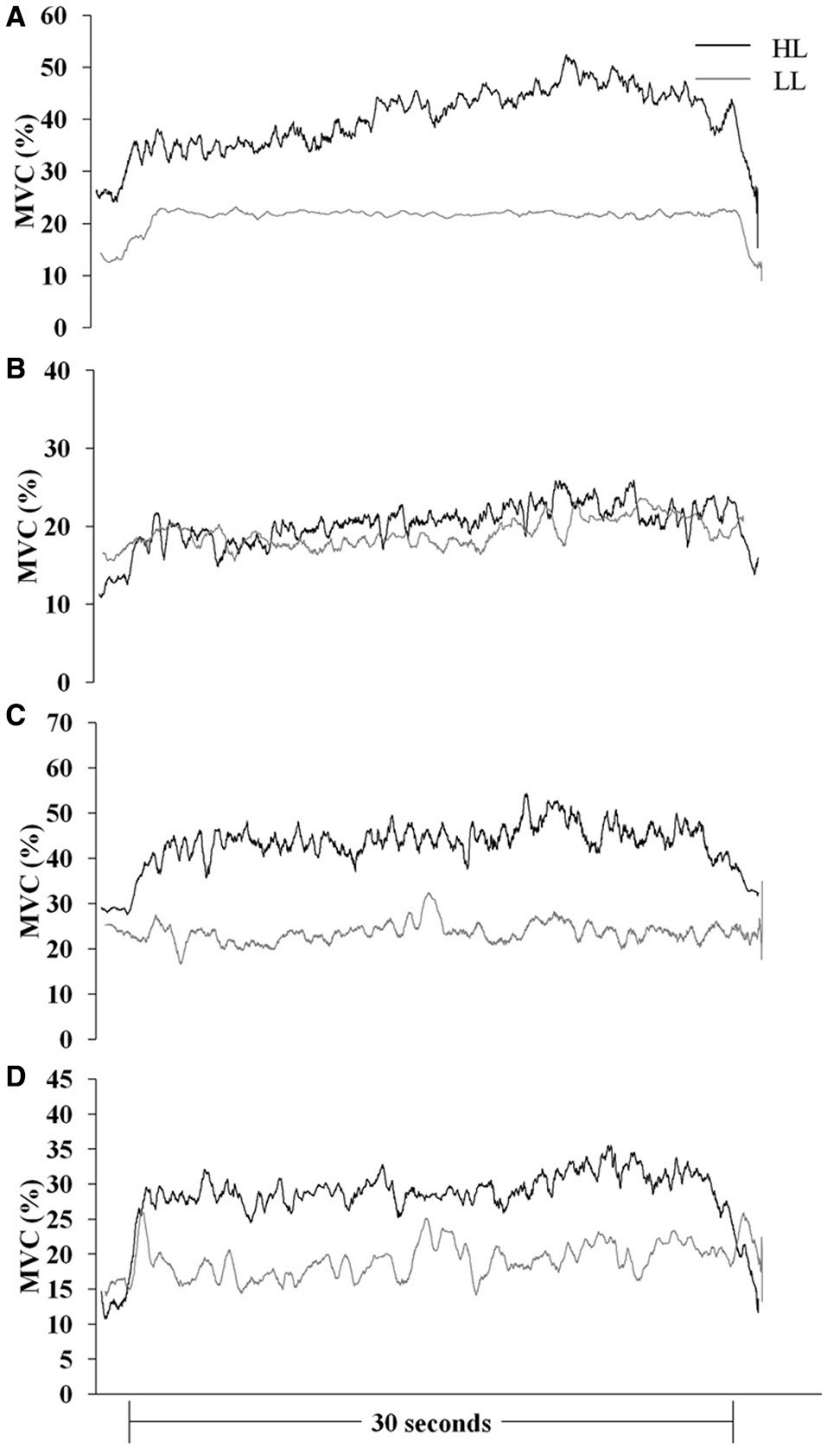
**Mean MVC-normalized surface electromyography root mean square (MVC %) recording over time during the HAL vibration intervention in international (HL)- and national (LL)-level groups. (A)** *brachioradialis*, **(B)** *extensor digitorum*, **(C)** *flexor carpi radialis*, and **(D)** *flexor digitorum superficialis*.

Muscle responses (sEMG_norm_ values) in BR, ED, FC, and FS during HAL and LAL are summarized in Table 1 and Figures 2, 3. The variability of the measurements ranged from low to medium values (CV 4÷20% in all the muscles). Statistical analysis revealed significant differences between the HL and LL groups in the sEMG of the ED muscle [*F*_(1, 24)_ = 20194.99, *P* < 0.001, η^2^ = 38.387, ES = large] with a significant interaction between the vibration intervention and TT level [*F*_(1, 24)_ = 10098.10, *P* < 0.001, η^2^ = 19.015, ES = large]. The BR muscle showed significant differences in the sEMG response between the two groups [*F*_(1, 24)_ = 53.92, *P* < 0.001, η^2^ = 0.154, ES = large], but the interaction between the vibration intervention and TT level was not significant [*F*_(1, 24)_ = 3.124, *P* = 0.072, η^2^ = 0.0094, ES = small]. In the FC muscle the sEMG response was higher in the LL than the HL group during LAL, whereas during HAL the sEMG response was higher in the HL than the LL group [*F*_(1, 24)_ = 5799.80, *P* < 0.0001, η^2^ = 28.941, ES = large]. In the FC muscle the interaction between the vibration intervention and TT level showed the highest effect size among the selected muscle [*F*_(1, 24)_ = 11737.23, *P* < 0.001, η^2^ = 62.096, ES = large]. In the FS muscle the difference in sEMG response between the two groups was the highest during LAL [*F*_(1, 24)_ = 1024.66, *P* < 0.001, η^2^ = 2.085, ES = large]; the interaction between the vibration intervention and TT level was also significant [*F*_(1, 24)_ = 11107.07, *P* < 0.001, η^2^ = 2.403, ES = large; Table 1]. HL revealed a significant change between HAL and LAL [(HAL-LAL)/LAL] in ED −7.27% (*P* < 0.001), BR −1.49% (*P* < 0.001), FC 13.36% (*P* < 0.0001), and FS −1.74% (*P* < 0.001). LL revealed a significant change between LAL and HAL in ED −39.61% (*P* < 0.001), BR −3.54% (*P* < 0.001), FC −48.47% (*P* < 00001), and FS 16.48% (*P* < 0.001).

**Table 1:** **Muscle responses during vibration interventions**.

**Muscle**	**Group**	**LAL**	**Δ% LAL ((HL-LL)/LL)**	**HAL**	**Δ% HAL ((HL-LL)/LL)**
ED (%max)	HL	45.01 ± 3.89	22.38^*^	41.74 ± 6.12	87.93^*^
	LL	36.78 ± 2.58		22.21 ± 0.87	
BR (%max)	HL	22.20 ± 2.25	4.72^*^	21.87 ± 2.81	6.94^*^
	LL	21.20 ± 3.33		20.45 ± 1.51	
FC (%max)	HL	40.11 ± 5.73	−22.73^*^	45.47 ± 6.26	69.98^*^
	LL	51.91 ± 4.57		26.75 ± 5.62	
FS (%max)	HL	30.53 ± 3.86	61.28^*^	30.00 ± 3.35	36.05^*^
	LL	18.93 ± 1.79		22.05 ± 1.46	

Surface electromyographic response root mean square normalized to maximal isometric voluntary contraction and relative change (Δ%) for each muscle between the two vibration interventions (low acceleration load [LAL] and high acceleration load [HAL]). Values are expressed as mean (percentage of maximum value of each muscle) ± SD for international level (HL) and national level (LL). Muscles' brachioradialis (BR), extensor digitorum (ED), flexor carpi radialis (FC), and flexor digitorum superficialis (FS) values are represented for LAL and HAL vibration interventions with

* *P < 0.0001 between HL and LL*.

## Discussion

The main findings of this investigation were that (1) international level (HL) table tennis (TT) players responded with greater muscle activation compared to national level (LL) table tennis players during low acceleration load (LAL) and high acceleration load (HAL) vibration interventions, except for the *flexor carpi radialis* (FC) during LAL; and (2) the vibration acceleration negatively affects muscle activity, particularly in the LL players. In fact, the muscle response differences between HL and LL increased during HAL with the exception of the *flexor digitorum superficialis* (FS) muscle in LL, in which its response tended to be higher during HAL than during LAL.

Previous studies have shown that an acute exposure to vibration increases neuromuscular and hormonal responses (Bosco et al., 1999; Mischi and Cardinale, 2009; Di Giminiani et al., 2013, 2014, 2015; Gyulai et al., 2013; Padulo et al., 2014). Specifically, Bosco et al. (1999) reported that national-level boxers exhibited an acute effect in mechanical power (isotonic cable forearm flexion) and surface electromyographic response root mean square (sEMG_rms_) of the *biceps brachii*. In addition, Mischi and Cardinale (2009) found greater muscle response in the *triceps brachii* and *biceps brachii* during vibration (vibrating dumbells) superimposed on extension-flexion exercises when compared to the non-vibration condition. In contrast, Cochrane and Hawke (2007) failed to show significant strength improvements (isometric handgrip test) following dumbbell vibration in the forearm muscles of climbers. Concerning the acute effect of direct vibration on the finger and wrist muscles (Martin and Park, 1997), found an increase in motor unit synchronization with increasing levels of force of up to 20% of the maximum voluntary contraction in the muscles exposed to vibrations with frequencies ranging from 40 to 200 Hz. They concluded that the vibration-induced increases in surface electromyographic (sEMG) response and the degree of motor unit synchronization were dependent on both the vibration frequency and the muscle contraction level. In our study, a higher sEMG response was observed in HL when compared to LL during both of the vibration interventions. Our results underscore that the degree of muscle activation is related to skill level. The higher activation of most of the target muscles in the HL compared to LL could be due to the greater increase in the motor unit recruitment (Ritzmann et al., 2010; Pollock et al., 2012) and voluntary drive (Mileva et al., 2009) when applying the vibrations.

In the present study, the exercise performed by the athletes (i.e., with isometric elbow flexion at 90°) was investigated, matching similar biodynamic responses in the kinetic chain system (wrist-metacarpal and finger-joint kinematics) commonly involved in TT skills. From a biomechanical perspective, TT skills are open kinematic chain movements with a proximal-distal sequence that have the function of accelerating the most distal segment (i.e., the racket). It seems to be advantageous for TT players to be able to accelerate the racket, because a limited time is allocated for executing the strokes during a match (Ak and Kocak, 2010). The fast motor unit recruitment may possibly be one of the factors contributing to greater speed at the time of impact with the ball. Moreover, the contraction speed of a muscle also affects its force magnitude, because the speed affects the force exerted by the muscle according to its force-speed relationship. Sakurai and Ohtsuki (2000) reported sEMG responses on the muscles that control wrist actions (the *extensor carpi radialis* and FC) in the 50 ms before the collision of the racket with the ball. They showed an extension-flexion-extension sequence of muscle activity that relates to the preparatory “cocking” movements of forearm supination-wrist extension-radial flexion, followed by the action movements of forearm pronation-wrist flexion-ulnar flexion that provide the power at impact. In a comparison of skill among players of different levels, they found that this sequence of muscle activity was well-defined and consistent in skilled players, but insignificant and inconsistent in unskilled players. Their results suggested that the unskilled players were not able to adequately control the final motion of the stroke before impact, and therefore their shot lost power. Previous studies suggest that an increase in sEMG response occurs when the excitation of the *alpha* motoneurons increases *via* the muscle spindle system during vibration exposure (Burke and Gandevia, 1995; Eklund and Hagbarth, 1966). In addition, the increased sEMG response could be attributable to changes in corticospinal excitability and intracortical processes. Mileva et al. (2009) found increased MEPs of the *tibialis anterior* during whole-body vibration, and Siggelkow et al. (1999) found that MEPs were increased in the *extensor carpi radialis* during localized muscle vibration at different acceleration loads.

In the present study, differences in sEMG response between the HAL and LAL interventions emphasize that the muscle activity patterns are modified as a response to changes in the excitation acceleration of input signals. In this context, Siggelkow et al. (1999) have reported changes in the corticospinal excitability, in which the augmentation of MEPs is sensitive to changes in vibration frequency. In addition, the observed vibration-induced effects in the forearm muscles showed a muscle-dependent pattern. Specifically, we found an inverse pattern for FC in HL and FS in LL, whose activations resulted higher during the HAL when compared to the LAL condition. Such findings can be explained by the evidence that the Ia-afferent activity that reinforces the recruitment of high-threshold motor units during direct vibration is clearly different for the tonic and the phasic muscles (Ushiyama et al., 2005). Additionally, the motor unit population supplying the vibrated muscle shows enhanced activity in the production of tonic vibration reflex (which mediates the motor unit recruitment) when increasing the tendon vibration frequency. That is, the increase in the vibration frequency does not modify the slow motor units' discharge pattern, but affects the fast motor units (Romaiguère et al., 1991). Therefore, the fiber-type proportions in the FC and FS muscles, relative to the variability between individuals (HL and LL; Schiaffino and Reggiani, 2011), could explain the differences in the muscular activation when applying various acceleration loads (HAL and LAL). Also, the variability in the mechanical characteristics of the muscles (i.e., cross-sectional area, length, etc.) among the subjects could determine differences in the muscle activity in order to minimize resonance when the vibration frequency is close to the natural frequency of the same muscle (Wakeling et al., 2002). It could also be useful to measure the dumbbell grip force, which could have an effect on sEMG_rms_. However, further investigations will be necessary in order to arrive at a conclusive picture and to state that muscle activation levels of the FC and FS are neurophysiologically- or mechanically-dependent.

## Conclusions

Our study confirms that indirect-localized vibration applied to the forearm muscles determines acute neuromuscular responses in relation to skill level of TT players. Future investigations should take in account these acute responses that could be used to induce specific adaptations during the training process. Specifically, the acceleration load plays an important role in maximizing the muscle activation in TT players of different skill level. Therefore, these results underline the importance of individualization of the acceleration load by using the sEMG response in the muscles that have the greatest involvement in each task. Particularly, it could be interesting to apply the optimal load and to monitor the vibration effect on the skill level of TT.

## Author contributions

All authors listed, have made substantial, direct and intellectual contribution to the work, and approved it for publication.

### Conflict of interest statement

The authors declare that the research was conducted in the absence of any commercial or financial relationships that could be construed as a potential conflict of interest.
